# A Case of Digital Papillary Carcinoma Arising on the Toe in a Young Female

**DOI:** 10.7759/cureus.63776

**Published:** 2024-07-03

**Authors:** Lily Park, Jennifer Wong

**Affiliations:** 1 Dermatology, Larkin Community Hospital, Miami, USA; 2 Department of Dermatology, Nova Southeastern University Dr. Kiran C. Patel College of Osteopathic Medicine, Fort Lauderdale, USA; 3 Dermatology, Aqua Dermatology, Weston, USA

**Keywords:** syringocystadenoma papilliferum on toe, digital papillary adenoma, syringocystadenocarcinoma papilliferum, aggressive digital papillary adenocarcinoma, syringocystadenoma papilliferum

## Abstract

Syringocystadenoma papilliferum is a rare benign adnexal hamartoma that is often associated with the nevus sebaceous of Jadassohn. It usually presents on the scalp and malignant transformation is rare. Here we present a case of digital papillary carcinoma on the toe of a teenage girl. The lesion recurred after two prior excisions without biopsy. The biopsy was read as a syringocystadenoma papilliferum with concerns for aggressive digital papillary adenocarcinoma, highlighting the importance of biopsy with excisions of neoplasms of unknown etiology.

## Introduction

Syringocystadenoma papilliferum (SCAP) is a rare benign adnexal hamartoma often associated with the nevus sebaceous of Jadassohn and appears almost exclusively on the head and neck [1-3]. SCAP typically presents as a solitary papule, nodule, or plaque. However, cases of multiple eruptions clustering together or organized linearly have also been documented [1,4,5]. Most lesions appear at birth or develop during childhood and are known to change in appearance after puberty. SCAP occurs more frequently in females, although there has not been an epidemiological study to confirm this [3]. Almost all lesions involve the head and neck, although emergence from other areas has also been described as well [2]. Our literature search on PubMed yielded only two previous cases of SCAP appearing on the toes, making ours the third reported case in this location [5,6].

SCAP is classically benign and painless; however, rare instances of its transformation into syringocystadenocarcinoma papilliferum, a rare malignant adnexal neoplasm, have occurred [7]. No reports of SCAP with concerns with aggressive digital papillary adenocarcinoma (ADPAca) were found in our literature search on PubMed. Our case presents a SCAP on the toe of a teenage female patient with the possibility of this malignant transformation.

## Case presentation

An 18-year-old Caucasian female with no pertinent past medical history presented with two lesions, one on the left lateral nipple and one on the left dorsal fifth toe. These two lesions had been present since the patient was 1 year of age and were noted to exhibit erythema, pruritus, and occasional pain. The patient reported having undergone two surgical excisions without a tissue biopsy.

**Figure 1:**
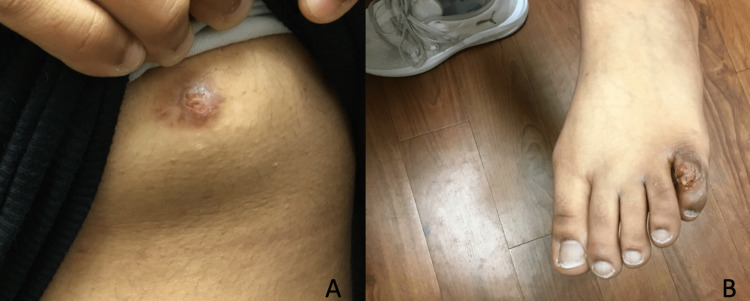
Clinical images of erosive adenomatosis of the nipple and syringocystadenoma papilliferum of the toe. Tan-brown exophytic papillary nodules with erosion, pinpoint bleeding, and mild crusting: on the left breast (A), and on the left fifth dorsal toe (B).

During a skin examination, a tan-brown exophytic papillary nodule with erosion, pinpoint bleeding, and crusting measuring 0.8 cm × 0.7 cm × 0.2 cm was observed on the left areola (Figure 1A). Similarly, the lesion on the left dorsal fifth toe exhibited a similar morphology, measuring 1.2 cm × 0.7 cm × 0.6 cm (Figure 1B).

**Figure 2:**
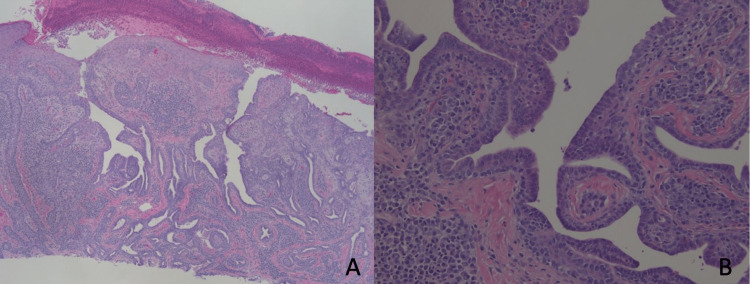
Histology of the left breast lesion. Ulceration seen on epidermis. Endophytic papillary projections are observed and glandular spaces are lined by two layers of columnar and apocrine cells displaying decapitation secretion. While the histologic features were identical to syringocystadenoma papilliferum, due to the anatomical location a diagnosis of nipple adenoma, or erosive adenomatosis was favored. Hematoxylin and eosin stain (H&E) 40× (A), 400× (B).

Deep shave biopsies were performed on the two lesions. Due to the complex histologic findings, the initial dermatopathology reading required reevaluation of the slides by a secondary dermatopathologist. After a careful analysis, the lesions on the left nipple were diagnosed as erosive adenomatosis, also known as nipple adenoma (Figures 2A-2B), and the lesions on the left fifth toe were diagnosed with SCAP, exhibiting features resembling syringocystadenocarcinoma papilliferum.

**Figure 3:**
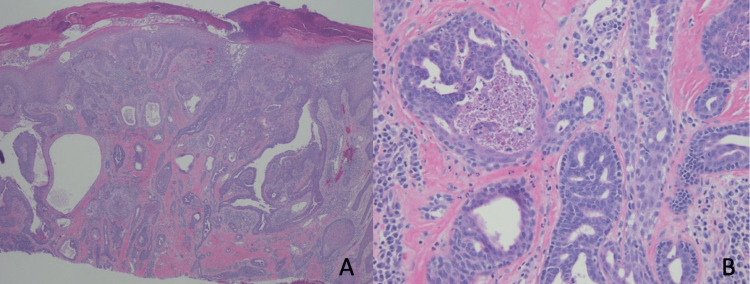
Histology of the left fifth toe lesion. Eroded epidermis overlying an endophytic glandular adnexal neoplasm with papillary projections. Compared to the histology of the left breast (Figures 2A-2B), there was a more pronounced cribriform growth pattern with increased nuclear hyperchromasia and areas of necrosis. H&E stain, 40× (A), 400× (B).

However, considering the anatomical location of the toes, pathologists reported that ADPA could not be ruled out (Figures 3A-3B).

Due to the esthetic concerns and the possibility of malignancy, the patient was referred to a plastic surgeon for the wide excision of both lesions with negative margins.

At the 1-year follow-up, the patient showed no signs of recurrence in either of the locations.

## Discussion

SCAP is predominantly found on the head and neck during the period between birth and childhood [1,2]. Although cases of multiple lesions have been reported [3], it typically manifests as a solitary papule, nodule, or plaque with a gray to dark brown color, often exhibiting a warty appearance, with crusting [4]. These lesions, particularly when solitary, are commonly observed on the head and neck and are frequently associated with the nevus sebaceous of Jadassohn [1]. Although less commonly, lesions appearing on other parts of the body, such as the trunk, extremities, and genitalia, have also been documented [1,3,5,6]. In rare instances where SCAP manifests as multiple lesions, they tend to merge and form plaques or clusters, and in exceptional cases, exhibit a linear pattern [6].

SCAP is associated with mutations in the mitogen-activated protein kinase signaling pathway, particularly in the *BRAF*, *KRAS*, and *HRAS* genes. It can occur sporadically but is more commonly found in the context of a cutaneous mosaic RASopathy, such as nevus sebaceous of Jadassohn [7].

The epithelial myoepithelial differentiation of SCAP can be detected using a set of immunostain markers. These include smooth muscle actin (SMA), calponin, SOX10, and p63 to highlight the outer myoepithelial layer, along with luminal cytokeratins and epithelial membrane antigen (EMA) for identifying the inner layer [8]. In our patient’s case, immunostaining was not used.

Histopathological examination is the primary diagnostic method for SCAP. It is characterized by wide papillary processes, or fronds, that connect the main body of the lesion to its surface squamous epithelium. These fronds are lined by a bilayer of cells with columnar apocrine cells displaying decapitation secretion on the luminal layer, and smaller cuboidal cells with larger nuclei and relatively scant amounts of cytoplasm on the outer layer. This bilayer forms a glandular ductal lining, resulting in a cribriform pattern. However, other arrangements such as lace-like patterns or cyst-like ducts can also be observed. The cores of these fronds typically contain a dense infiltrate of plasma cells and lymphocytes [1,4].

When distinguishing SCAP from nipple adenoma, SCAP is characterized by an inward-growing, crater-like lesion with a significant plasmacytic infiltrate in the stroma. A surface connection is a key feature of SCAP, although it can also appear in nipple adenoma. However, in nipple adenoma, this surface connection is generally more localized and less pronounced compared to the extensive cystic invaginations observed in SCAP. Furthermore, nipple adenoma exhibits a more pronounced proliferation of tubular structures [9].

Although a double-layered epithelium with papillary proliferation is similarly observed in both diagnoses, apocrine metaplasia with decapitation secretion is uncommon in nipple adenoma compared with SCAP. In addition, squamous metaplasia and keratin-filled cysts that can be observed in SCAP are thought to be less frequently found in nipple adenoma. If those are found in nipple adenoma, they tend to be present only in the superficial portion [10].

SCAP has been documented to undergo malignant transformation into syringocystadenocarcinoma papilliferum (SCAPca) in extremely rare instances. A literature review by Zhang et al. revealed that most cases arose from pre-existing long-standing SCAP lesions, although they can also develop de novo, and were more prevalent among older patients, with a mean age of 63.6 [4,7,11].

The clinical presentation of SCAPca can be similar to that of SCAP. It appears as skin color to hyper-pigmented, exophytic plaque, nodule, or tumor with crust, which is due to the apocrine gland decapitation secretion [11]. Histopathologically, SCAPca shares similarities with SCAP, including cystic and papillary growths of epithelium, decapitation secretion with the inner columnar cell layer, and inflammatory infiltrates, with plasma cells. However, SCAPca differs from SCAP in its asymmetry, more extensive layers of invagination, high nuclear-to-cytoplasmic ratio, increased mitotic activities, and deep extension into the subcutaneous fat. Additionally, SCAPca may present with psammoma bodies, perineural invasion, pagetoid spread, squamatization, and sarcomatous changes. Although there are no specific immunohistochemical markers for SCAPca, cytokeratin 5/6, p63, and D2-40 staining with positive results tend to be associated with cutaneous neoplasms such as SCAPca instead of metastatic adenocarcinoma. SCAPca is thought to be a low-grade malignancy with a favorable outcome; however, there are no treatment protocols due to the rarity of this malignancy. Wide local excisions are the current treatment of choice although other modalities such as Mohs micrographic surgery, lymph node biopsy, chemotherapy, and radiation have been documented in case reports [12].

ADPAca is a rare variant of eccrine gland carcinoma that occurs nine times more commonly in males than in females, with a median age of occurrence at 52 years old [9]. Despite its name, ADPAca is considered a low-grade malignant tumor and can develop on acral surfaces [13]. The term “aggressive” refers to its tendency for intractable local recurrence if inadequately excised [1,13].

ADPAca has a metastatic potential of 14%, spreading hematogenously to the lung parenchyma and lymph nodes. Currently, there is no FDA-approved systemic treatment available [14]. Surgical excision is the current gold standard, although amputation of the affected digit may be necessary. SCAP is also treated primarily by surgical excision [1]. In this unique instance where ADPAca potentially masqueraded under the benign appearance of SCAP, a full-thickness excision of this lesion was performed, with biopsy and histologic evaluation, to ensure complete removal. Furthermore, the patient will be closely monitored by her primary care physician for any signs of lung and lymph node metastasis in the long term [1,15].

## Conclusions

Our case highlights important considerations that extend beyond the field of dermatology and should be taken into account by all physicians. The delayed biopsy of the lesions, despite their long-standing presence since the patient’s infancy, possibly resulted in recurrence following multiple incomplete excisions performed without biopsies. As physicians, we should recognize the significant implications of a skin biopsy, particularly in the context of potential malignancy. The histopathological features in our patient’s lesions exhibited similarities with ADPAca. Moreover, our case underscores that SCAP can manifest in uncommon locations, such as the extremities. Having this knowledge would allow us to expand our differential diagnoses, which would help us get to the correct diagnosis for optimal management.

## References

[1] McCalmont TH (2012). Adnexal neoplasms. Dermatology.

[2] Ghazeeri G, Abbas O (2014). Syringocystadenoma papilliferum developing over hyperkeratosis of the nipple in a pregnant woman. J Am Acad Dermatol.

[3] Requena L, Sangüeza O (2017). Syringocystadenoma Papilliferum. Cutaneous Adnexal Neoplasms.

[4] Brenn T (2020). Tumors of the Sweat Glands. McKees Pathology of the Skin: With Clinical Correlations.

[5] Nobeyama Y, Ito Y, Nakagawa H (2013). Syringocystadenoma papilliferum on the dorsum of the foot. J Dermatol.

[6] Patterson JW, Straka BF, Wick MR (2001). Linear syringocystadenoma papilliferum of the thigh. J Am Acad Dermatol.

[7] Aslam A, Salam A, Griffiths CE, McGrath JA (2014). Naevus sebaceus: A mosaic RASopathy. Clin Exp Dermatol.

[8] Macagno N, Sohier P, Kervarrec T, Pissaloux D, Jullie ML, Cribier B, Battistella M (2022). Recent Advances on Immunohistochemistry and Molecular Biology for the Diagnosis of Adnexal Sweat Gland Tumors. Cancers.

[9] Weigelt MA, Sciallis AP, McIntire PJ, Ko JS, Billings SD, Ronen S (2023). Nipple adenoma: Clinicopathologic characterization of 50 cases. Am J Surg Pathol.

[10] Kasashima S, Kawashima A, Fujii T (2016). Syringocystadenoma papilliferum of the male nipple. J Cutan Pathol.

[11] Zhang YH, Wang WL, Rapini RP, Torres-Cabala C, Prieto VG, Curry JL (2012). Syringocystadenocarcinoma papilliferum with transition to areas of squamous differentiation: A case report and review of the literature. Am J Dermatopathol.

[12] Lee KG, Choi W, Lim JS, Hahn HJ, Myung KB, Cheong SH (2019). Syringocystadenocarcinoma papilliferum: A case report and review of the literature. Ann Dermatol.

[13] Chen S, Asgari M (2014). Is aggressive digital papillary adenocarcinoma really aggressive digital papillary adenocarcinoma?. Dermatol Pract Concept.

[14] Altmann S, Damert HG, Klausenitz S, Infanger M, Kraus A (2015). Aggressive digital papillary adenocarcinoma - a rare malignant tumor of the sweat glands: Two case reports and a review of the literature. Clin Cosmet Investig Dermatol.

[15] Losch A (2009). Congenital linear syringocystadenoma papilliferum mimicking nevus sebaceous. J Am Acad Dermatol.

